# Phosphorus Deficiency Affects Memory‐Mediated Recovery From Recurrent Water Stress in Drought‐Sensitive Soybean

**DOI:** 10.1111/ppl.70772

**Published:** 2026-02-04

**Authors:** Isadora Rodrigues Medina Santana, Guilherme Henrique da Rocha, Gabriela Píccolo Maitan‐Alfenas, Eduardo Gusmão Pereira

**Affiliations:** ^1^ Instituto de Ciências Biológicas e da Saúde, Universidade Federal de Viçosa (UFV) Florestal Brazil; ^2^ Departamento de Bioquímica e Biologia Molecular, UFV Viçosa Brazil

**Keywords:** fatty acid profile, *Glycine max* L., mesophyll conductance, phosphorus nutrition, photosynthetic limitation, water deficit

## Abstract

Phosphorus (P) deficiency and water deficit are major constraints to soybean yield worldwide. While their individual impacts are well established, little is known about how P deficiency modulates soybean recovery from recurrent water stress. This study evaluated the effects of P deficiency on the recovery capacity of two soybean cultivars, contrasting in drought sensitivity, during the grain‐filling stage. Plants were grown under either high P availability or P deficiency and subjected to different irrigation regimes: well‐watered (WW), severe water deficit at R5 (WS‐R5), and moderate deficit at V5 followed by severe deficit at R5 (WS‐V5 + R5). The experiment followed a randomized complete block design in a 2 × 3 factorial scheme. Under water stress, P deficiency delayed stomatal resistance, extending photosynthetic decline in both cultivars. However, recovery of photosynthetic rate and stomatal conductance was faster under P deficiency than under high P supply. In the sensitive cultivar, P deficiency enhanced memory‐mediated recovery of photosynthesis only after two stress cycles, with compensatory increases in mesophyll conductance, decreasing mesophyll limitations and favoring recovery. In contrast, the tolerant cultivar showed stable photosynthetic responses regardless of P level, with similar recovery in light saturation and photorespiration. Grain composition was affected by P deficiency in both cultivars, with lower protein concentration and increased oil content, particularly of unsaturated fatty acids. These results indicate that P deficiency alters physiological adjustments in soybean genotypes sensitive to water deficit, influencing their capacity to recover from recurrent drought stress and affecting grain quality.

## 1 Introduction

Soybean ( *Glycine max* L.) is the most important legume crop globally, mainly used as a source of oil and protein for animal feed (Sentelhas et al. 2015). Brazil is the world's top producer, with 47,6 million hectares under cultivation (EMBRAPA 2025). In the 2023/2024 season, soybean production was estimated at 155.3 million tons, down 4.2% from the initial forecasts of 162 million tons. Recent environmental changes, primarily poorly distributed rains and high temperatures, are the main causes of yield loss in soybean farming, especially in rainfed systems (He et al. 2020).

Water deficit impacts soybean morphophysiology throughout its development. When stress occurs during vegetative stages, tolerant plants can sustain grain production through adaptive mechanisms (Medina et al. 2023). However, water demand doubles during reproduction (Poudel et al. 2023), and stress at this stage hampers flower set, seed size, weight, and composition, ultimately decreasing yield (Du et al. 2020; Mesquita et al. 2020). Photosynthetic CO_2_ assimilation is limited by stomatal and non‐stomatal factors (Pilon et al. 2018; Song et al. 2020). Non‐stomatal effects include excessive reactive oxygen species (ROS) production (Kaur and Asthir 2017), which damages membranes and increases malondialdehyde (MDA) levels (Zhou et al. 2022).

Upon rehydration, photosynthesis and growth may resume, but the extent of recovery depends on the stress intensity and duration (Fleta‐Soriano and Munné‐Bosch 2016). Severe stress often leads to only partial recovery, whereas moderate stress can trigger compensatory growth (Dong et al. 2019), an essential self‐regulatory mechanism (Niu et al. 2018). Moreover, recovery from initial stress can prime plants for subsequent events through metabolic memory, improving tolerance to recurrent water deficits (Crisp et al. 2016; Auler et al. 2021; Sintaha et al. 2022). Memory‐mediated responses, such as improved photosynthetic adjustment and proline accumulation, have been reported in soybean (Medina et al. 2023), enabling grain production under recurrent drought.

However, nutrient imbalances, particularly phosphorus (P) deficiency, may hinder these mechanisms. P is essential for energy metabolism, carbon assimilation, and stress responses (Jin et al. 2007). P deficiency decreases soybean yield and alters grain quality by modifying fatty acid composition (Krueger et al. 2013; Win et al. 2010). While the independent effects of P deficiency and water deficit are well documented (Malhotra et al. 2018; Seleiman et al. 2021; Lazali and Drevon 2021), their interaction during recovery from recurring stress remains unclear. Since energy demand for stress recovery is closely related to P metabolism, deficiencies may hinder the development of stress memory and limit photosynthetic resilience (Gelaw et al. 2023).

Therefore, it is crucial to understand how P deficiency influences memory‐mediated recovery from recurrent water deficit in soybean. This study aimed to elucidate the impact of P deficiency on photosynthetic recovery, grain yield, and fatty acid profile in cultivars with contrasting drought tolerance. We hypothesize that tolerant cultivars will show faster memory activation under recurrent stress, while high P supply will enhance photosynthetic recovery, decrease oxidative damage, and mitigate yield and quality losses.

## 2 Material and Methods

### 2.1 Plant Species and Growth Conditions

The experiment was conducted in a greenhouse at the Federal University of Viçosa Campus Florestal (19°52′35.1″S 44°24′49.6″W). The average temperature and relative humidity inside the greenhouse were 26.4°C and 40.5%, respectively. We used the soybean ( *Glycine max* L.) cultivars EMBRAPA048 and TMG7063, which are known for being tolerant and sensitive to drought, respectively (Freitas et al. 2019; Winck et al. 2023). Both cultivars belong to the early maturity group; however, EMBRAPA048 and TMG7063 have determinate and indeterminate growth habits, respectively (Higashi et al. 1999). The soybean seeds were treated with 250 mL 100 kg^−1^ Vitavax Thiram fungicide (carboxanilide + dimethyldithiocarbamate) and inoculated with 60 g 100 kg^−1^ Adhere. Then, 5 soybean seeds per pot were sown (20 dm^3^), with a substrate composed of soil and sand in a 3:1 (v:v) ratio.

After emergence, thinning was performed, leaving only one plant per pot. The soil used has physical characteristics similar to dystrophic red latosol, with initial values of 0.49 g kg^−1^ total nitrogen, 2.4 mg dm^−3^ phosphorus, 65 mg dm^−3^ potassium, 0.62 cmolc dm^−3^ calcium, 0.21 cmolc dm^−3^ magnesium, 1.34 cmolc dm^−3^ aluminum, 3.5 cmolc dm^−3^ total acidity, 1.0 cmolc dm^−3^ sum of bases, 2.34 cmolc dm^−3^ effective cation exchange capacity, 4.5 cmolc dm^−3^ total cation exchange capacity, 22.2% base saturation, 57.3% aluminum saturation, 7.7 mg L^−1^ remaining phosphorus, and pH 4.66. The pH was corrected with dolomitic limestone, PRNT 80%, in the quantity obtained by the base saturation method (3.78 t ha^−1^), considering soil chemical analysis. Additionally, the limestone quantity was sufficient to meet the Ca^2^+ and Mg^2^+ requirements and to stimulate nitrogen‐fixing bacterial activity. Potassium oxide fertilization was applied at a dosage of 40 kg ha^−1^. Two treatments were set for P availability: high phosphorus (HP) and low phosphorus (LP). Phosphorus fertilization was applied only to pots from the HP treatment, at a rate of 120 kg ha^−1^ (Ribeiro et al. 1999), the maximum recommended dose for the soil type.

Three water deficit conditions were imposed: (1) WW, well‐watered (control condition); (2) WS‐R5, severe water deficit at R5; (3) WS‐V5 + R5, moderate water deficit at V5 and severe at R5. Soil moisture was monitored with tensiometers, maintaining it at field capacity (around 30 KPa) throughout the crop cycle in control well‐watered plants (WW) and until V5 (Fehr and Caviness 1977) in the plants exposed to water stress (WS). At the V5 stage, plants in the WS‐V5 + R5 treatment were subjected to a moderate water deficit, maintained for 2–3 days until the soil water tension, as assessed by tensiometers, reached 80 kPa. Then, the soil was irrigated and maintained at field capacity. At the beginning of the grain‐filling stage (R5), the plants from the WS‐R5 treatment, and again the WS‐V5 + R5 treatment, were subjected to severe water deficit. Plants were kept without irrigation until net photosynthesis reached near zero, indicating severe stress, which lasted from 4 to 6 days in plants from the HP treatment and from 9 to 14 days in plants from LP treatment. Therefore, water deficit was restricted to the V5 and R5 stages. After this period, irrigation was resumed, and the recovery of photosynthetic metabolism was monitored. There was no water restriction in the control treatment (WW) throughout the crop cycle.

### 2.2 Experimental Design and Statistical Analysis

A randomized complete block design was adopted in a 2 × 3 factorial, with two substrate conditions (high and low phosphorus availability) and three irrigation conditions (WW—well‐watered; WS‐R5—severe water deficit at R5; WS‐V5 + R5—moderate water deficit at V5 and severe at R5), with five replications.

The data were considered homogeneous and normal, using the Barlett and Shapiro–Wilk tests, respectively. Subsequently, they were subjected to analysis of variance (ANOVA) and compared using the Tukey test (*p* < 0.05), carried out in the statistical program RStudio (version 3.6.3).

### 2.3 Monitoring and Characterization of Severe Water Stress

#### 2.3.1 Gas Exchange

Gas exchange assessments were conducted daily, starting from the onset of severe water deficit imposition at the R5 stage. Net photosynthetic rate (*A*_n_) and stomatal conductance (*g*_s_) measurements were taken in the morning with the first fully expanded leaf from the apex, on the central leaflet. An infrared gas analyzer (LI‐6400xt, Li‐Cor Inc.) was used with a photosynthetic photon flux density (PPFD) of 1200 μmol m^−2^ s^−1^, CO_2_ concentration of 400 μmol mol^−1^, temperature of 28°C, and relative humidity of 44%.

#### 2.3.2 Water Potential

Predawn leaf water potential (ψ_w_) was measured in the second mature leaf, using a digital Scholander pressure chamber, model 1400/80 (PMS Instrument Company), on the last day of severe water stress at the R5 stage.

#### 2.3.3 Proline

Proline concentration was determined in the second mature leaf collected on the last day of severe water stress, following the protocol of Bates et al. (1973).

#### 2.3.4 Determination of Malondialdehyde

Malondialdehyde (MDA) levels were estimated using the methodology proposed by Hodges and DeLong (1999), in the first mature leaf, collected on the last day of severe water stress and stored in liquid nitrogen until analysis.

#### 2.3.5 Determination of Hydrogen Peroxide (H_2_O_2_ )

The H_2_O_2_ concentrations were estimated according to the methodology proposed by Velikova et al. (2000), in the first mature leaf, collected on the last day of severe water stress and stored in liquid nitrogen until analysis.

### 2.4 Monitoring and Characterization of Water Stress Recovery

#### 2.4.1 Light Saturation Curve (*A*_n_/PPFD) and CO_2_ Saturation Curve (*A*/*Ci*)

During the recovery period with resumed irrigation, gas exchange assessments were conducted as previously described, using the LI‐6400xt infrared gas analyzer (Li‐Cor Inc.), equipped with a fluorescence chamber (Li‐6400‐40). The water status was considered fully recovered only when the plants exhibited a net photosynthetic rate statistically similar to that of the control plants.

After the completion of this rehydration process, light saturation (*A*_n_/PPFD) and CO_2_ saturation (*A*_n_/*Ci*) curves were performed on the central leaflets of mature leaves. Light saturation measurements were taken at 2000, 1500, 1250, 1000, 800, 600, 400, 200, 100, 75, 50, 25, 0 μmol photons m^−2^ s^−1^. The minimum pre‐established time for stabilization of readings at each light radiation level was 60 s. Atmospheric CO_2_ inside the leaf chamber was kept constant at 400 μmol CO_2_ mol^−1^ during the light‐saturation curve determinations. The data were adjusted according to Lobo et al. (2013) and Smith (1936), according to Equation (1):

(1) $$A_{n}=\left[\phi \;\left(\mathrm{Io}\right)\times I\times P_{g}\max/{\left(\phi \;{\left(\mathrm{Io}\right)}^{2}\times I^{2}+P_{g}\max^{2}\right)}^{0.5}\right]-R_{D}$$

where

- *A*_n_ = net photosynthetic rate [μmol (CO_2_) m^−2^ s^−1^];
- ϕ (Io) = quantum yield at light saturation point I = 0 [mmol (CO_2_) mmol^−1^ (photons)];
- I = photosynthetic photon flux density [μmol (photons) m^−2^ s^−1^];
- *P*_g_max = maximum gross photosynthesis rate [μmol (CO_2_) m^−2^ s^−1^];
- *R*_D_ = dark respiration rate [μmol (CO_2_) m^−2^ s^−1^].

From this modeling, values of maximum photosynthetic rate (*A*_max_), light compensation point (*I*_comp_), light saturation point beyond which there is no significant change in net photosynthesis (*I*_max_), and effective quantum yield at the compensation point (ϕ (*I*_comp_)).

The CO_2_ saturation curves were conducted at levels of 400, 300, 200, 100, 50, 400, 500, 600, 700, 800, 1000, 1200, and 1300 μmol CO_2_ mol^−1^. The minimum time for stabilization of readings at each concentration was 60 s and a maximum of 120 s. The data obtained in the *A*_n_/*Ci* curve were used to calculate the Rubisco carboxylation rate (*V*_cmax_), electron transport rate (*J*), triose phosphate utilization (*TPU*) (Sharkey et al. 2007). Mesophyll conductance (*g*_m_, mol CO_2_ m^−2^ s^−1^) and chloroplastidic CO_2_ concentration (*C*_c_, μmol CO_2_ mol^−1^) were estimated using the method described by Harley et al. (1992), according to Equations (2) and (3):

(2) $$g_{m}=A_{n}/\left(\mathrm{Ci}-\;\left(\Gamma *\left[J+8\;\left(A_{n}+R_{I}\right)\right]\right)/\left(J-4\;\left(A_{n}+R_{I}\right)\right)\right)$$

(3) $$C_{c}=\mathrm{Ci}-A_{n}/g_{m}$$

where *R*_I_ is respiration occurring during the day and not related to photorespiration. The value of the CO_2_ compensation point related to *Ci* (Γ* μmol mol^−1^) for soybean was calculated by the intersection of *A*_n_/*Ci* according to Walker and Ort (2015). From these, stomatal limitations (*l*_s_), mesophyll limitations (*l*_m_), and metabolic limitations (*l*_b_) were calculated according to the method described by Grassi and Magnani (2005), according to Equations ((4), (5), (6)):

(4) $$l_{s}\;\left(25^{{}^{\circ}}C\right)=\left(\left(g_{\mathrm{tot}}/g_{\mathrm{sc}}\right)*\left(ꝺA_{n}/ꝺC_{c}\right)\right)/g_{\mathrm{tot}}+ꝺA_{n}/ꝺC_{c}$$

(5) $$l_{m}\;\left(25^{{}^{\circ}}C\right)=\left(\left(g_{\mathrm{tot}}/g_{m}\right)*\left(ꝺA_{n}/ꝺC_{c}\right)\right)/g_{\mathrm{tot}}+ꝺ\;A_{n}/ꝺC_{c}$$

(6) $$l_{b}\;\left(25^{{}^{\circ}}C\right)=g_{\mathrm{tot}}/g_{\mathrm{tot}}+ꝺA_{n}/ꝺC_{c}$$

$$l_{s}+l_{m}+l_{b}=1$$

where *g*_tot_ is total conductance to CO_2_ between the leaf surface and carboxylation sites (1/*g*_tot_ = 1/*g*_sc_ + 1/*g*) and *g*_sc_ is stomatal conductance to CO_2_ (*g*_sc_ = *g*_s_/1.6).

The photorespiration rate (*P*_r_) was estimated using the following formula (Valentini et al. 1995), according to Equation (7):

(7) $$\mathrm{Pr}=\frac{1}{12}\left[\;\mathrm{ETR}-4\left(A_{n}+R_{D}/2\right)\right]$$

where ETR is the apparent rate of electron transport, obtained simultaneously with gas exchange analysis.

#### 2.4.2 Chlorophyll *a* Fluorescence

Chlorophyll *a* fluorescence was measured using the Mini‐PAM pulse‐amplitude‐modulated fluorometer (Heinz Walz). The evaluation took place at the end of the water stress recovery period in the morning, on the first fully expanded leaf from the apex, on the central leaflet. Minimum fluorescence (*F*_0_) and maximum fluorescence (*F*_m_) were measured in the early morning after leaf acclimation in the dark for at least 30 min. The obtained values were used to determine the maximum quantum efficiency of photosystem II (*PSII*; *F*_v_/*F*_m_) according to Equation (8):

(8) $$F_{v}/F_{m}=\left(F_{m}-F_{0}\right)/F_{m}$$

#### 2.4.3 Chlorophyll Indices

The total chlorophyll index was assessed at the end of the water‐stress recovery period using a portable ClorofiLOG meter (Falker). The index was characterized by the average of three measurements taken on the central leaflet of the first fully mature leaf from the apex.

### 2.5 Morphological Evaluations

At stage R8 (full pod maturity) (Fehr and Caviness 1977), the plants were partitioned into root, grain, pod, leaf, and stem parts, then dried in a forced‐air oven for 72 h at 65°C to determine dry biomass.

Leaf area (LA; cm^2^) was calculated according to Equation (9):

(9) LA=A×C×L

where *C* is the length of the leaflet (cm), *L* is the widest width of the leaflet (cm), *A* is the angular coefficient, where the value of 2.0185 was adopted (Richter et al. 2014).

### 2.6 Leaf P Concentration

The P concentrations were estimated according to the methodology proposed by Teixeira et al. (2017). Mature leaves were collected at the end of the water stress recovery period and also at the end of the biological cycle in R8 to quantify P concentration.

### 2.7 Oil and Protein Concentration in Grains

Oil extraction was carried out on 2 g of ground and dried grain samples using the Soxhlet method (Instituto Adolfo Lutz Institute 2008). The solvent used was petroleum ether. The percentage of lipids was given by the Equation (10):

(10) $$\text{Lipids}\;\left(\%\right)=\left(\mathrm{PL}\times 100\right)/P$$

where

- PL = weight of the balloon with fat—weight of the balloon before extraction.
- *P* = sample weight

For protein concentration, the Kjeldahl method was used, determining the total nitrogen (N) of the sample and later converting to crude protein using the factor 6.25. Four 0.5 g samples of dry, ground material were used for each cultivar in each test.

### 2.8 Grain Fatty Acid Profile

Fifteen milligrams of seed samples were ground and mixed with 1 mL of hexane for 16 h. Then, the solvent was evaporated under N_2_ and 0.4 mL of 1 M sodium methoxide was added, and the sample was incubated in a water bath at 30°C for 1 h. Subsequently, 1 mL of Milli‐Q water and 1 mL of hexane were added, and the sample was allowed to equilibrate for another hour. Finally, 0.75 mL of the organic phase was collected, sodium sulfate was added, and chromatographic reading was performed.

Analyses were carried out using a gas chromatograph model CG Solution by SHIMADZU, equipped with a FID detector. For chromatogram recording and analysis, the device is connected to a microcomputer using the GC Solution program. Compounds were separated and identified using a Carbowax capillary column (30 m × 0.25 mm).

For chromatographic separation, 1 μL of sample was injected using a 10 μL syringe (Hamilton) in Split = 5 system. Nitrogen gas was used as the carrier, with a linear velocity programmed to 43.2 cm/s, and hydrogen and synthetic air were used to form the flame in the detector. The injector and detector temperatures were controlled isothermally at 200°C and 220°C, respectively. The initial column temperature was 100°C (maintained for 5 min), then increased at 4°C per minute to 220°C (kept for 20 min). The carrier gas flow rate in the column was 1.0 mL/min.

## 3 Results

**FIGURE 1:**
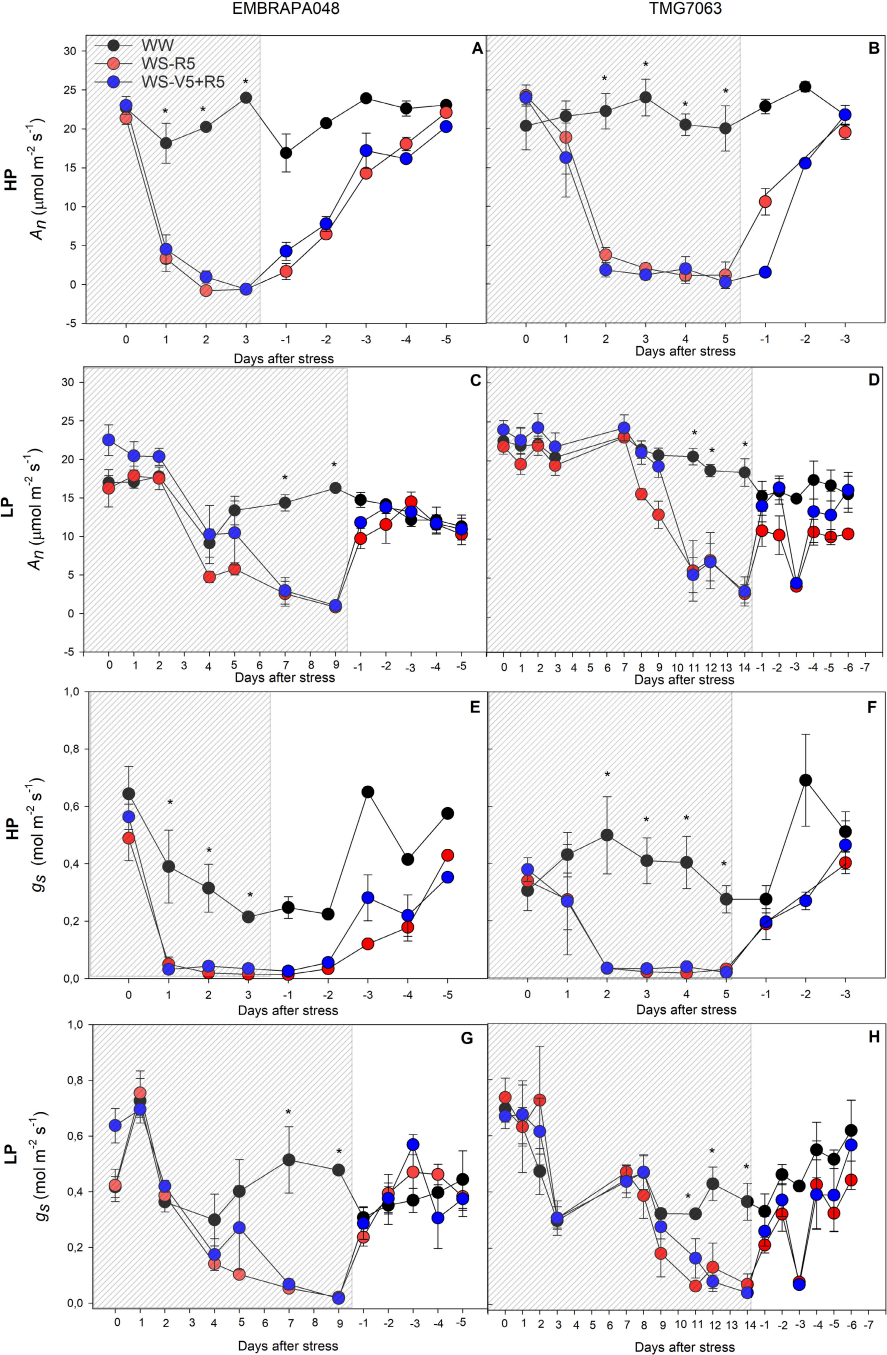
Net photosynthetic rate (*A*_n_; A, B, C, D) and stomatal conductance (*g*_s_; E, F, G, H) of soybean cultivars EMBRAPA048 (A, C, E, G) and TMG7063 (B, D, F, H) under high P conditions (HP) or P deficiency (LP), during water deficit in grain filling. The shaded area indicates the water stress period, and the white area indicates recovery after hydration. Symbols show the mean of five replicates, and error bars represent the standard error of the mean. Asterisks indicate statistical differences between treatments, according to Tukey's test at a 5% confidence level.

Regardless of the P nutritional condition, plants of both cultivars subjected to moderate water deficit at the V5 stage and subsequently exposed to severe stress at the R5 stage (WS‐V5 + R5) showed no differences in photosynthetic responses compared with those exposed solely to severe stress at the R5 stage (Figure 1).

The P deficiency prolonged the decline in photosynthesis (Figure 1C,D) and stomatal closure (Figure 1G,H) in response to water stress in both cultivars. However, the recovery of photosynthetic rates and stomatal conductance was faster in soybean plants under P deficiency than under high P conditions (Figure 1).

**FIGURE 2:**
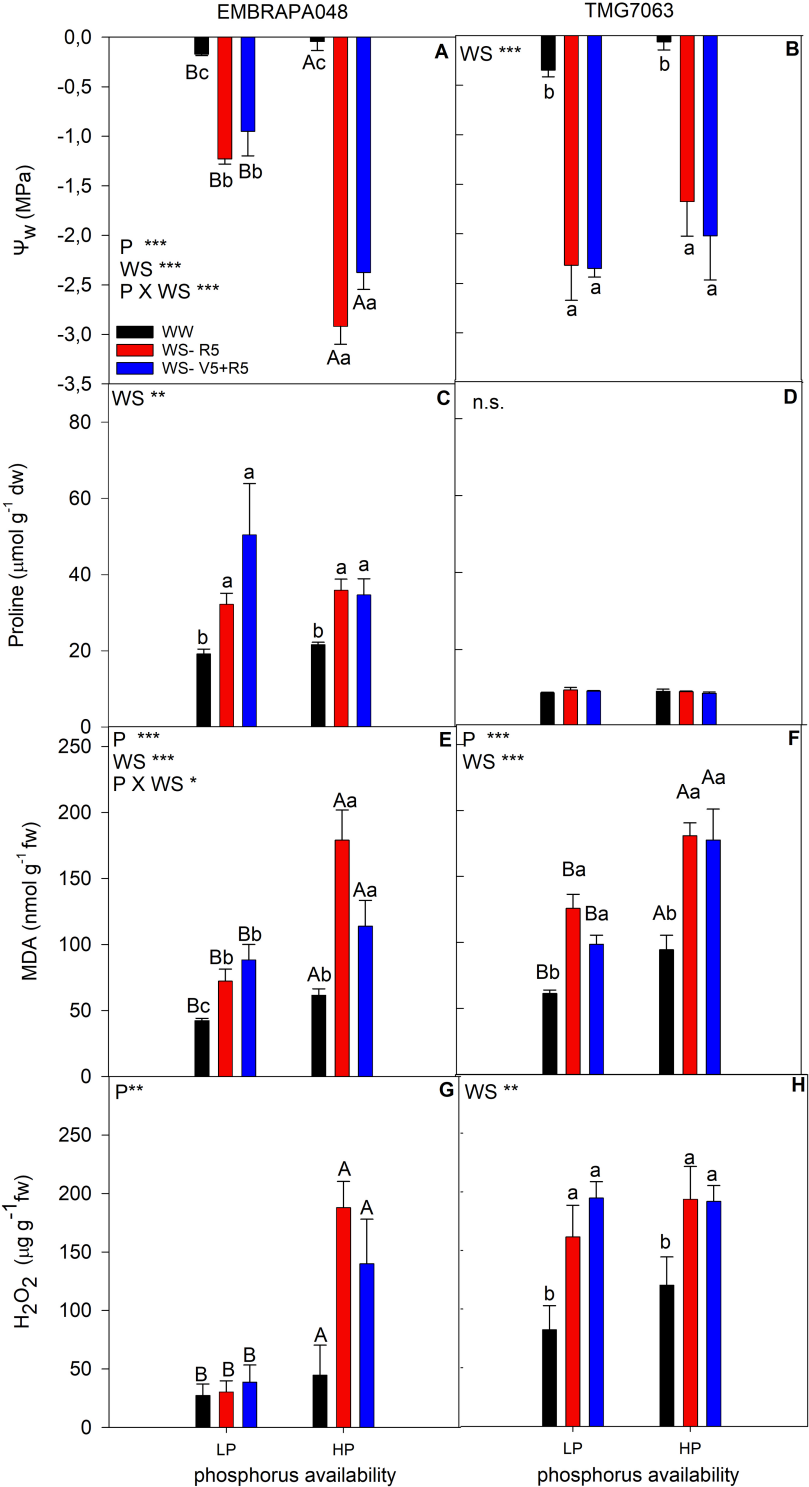
Leaf water potential (ψ_w_; A, B), proline (C, D), malondialdehyde (MDA; E, F), hydrogen peroxide concentration (H_2_O_2_; G, H) in soybean cultivars EMBRAPA048 (A, C, E) and TMG7063 (B, D, F) on the last day of severe water stress period under high P conditions (HP) or P‐deficient conditions (LP). Bars represent the mean of five replicates, and error bars represent the standard error of the mean. Only when significant by the ANOVA, the uppercase letters indicate statistical differences between nutritional conditions, and lowercase letters indicate statistical differences between water conditions, determined by Tukey's test at the 5% confidence level.

Both cultivars exhibited lower leaf water potential due to water stress (Figure 2A,B). However, only the cultivar EMBRAPA048 maintained higher leaf water potential under water deficit and phosphorus deficiency conditions (Figure 2A) than plants with higher P availability. The drought‐tolerant cultivar, EMBRAPA048, also accumulated higher proline concentration in leaves when exposed to both WS‐R5 and WS‐V5 + R5 treatments (Figure 2C). However, there was no significant difference in proline concentration in cultivar TMG7063 during drought stress (Figure 2D).

The MDA concentration in leaves was higher in plants subjected to water stress and to higher P availability in both cultivars (Figure 2E,F). The cultivar EMBRAPA048 exhibited higher H_2_O_2_ concentration under higher P availability than under LP treatments (Figure 2G). In contrast, the TMG7063 cultivar showed higher H_2_O_2_ levels in response to both water deficit conditions (WS‐R5 and WS‐V5 + R5), regardless of the P availability (Figure 2H).

Recovery from water deficit did not affect *A*_max_ and *I*_max_ in both cultivars; a decrease in these variables only occurred with P deficiency (Table 1). Both cultivars showed no difference in light compensation irradiance under either water or P conditions. However, the effective quantum yield at the compensation point (ϕ(*I*_comp_)) was lower in soybean plants under P deficiency. In both cultivars, *F*_v_/*F*_m_ and the total chlorophyll index were higher in soybean plants under high P conditions than in those under P deficiency (Table 1).

**TABLE 1:** Maximum photosynthetic rate (*A*_max_), light compensation point (*I*_comp_), light saturation point beyond which there is no significant change in net photosynthesis (*I*_max_), effective quantum yield at the compensation point (ϕ (*I*_comp_)), of soybean cultivars EMBRAPA048 and TMG7063 during the recovery period from water stress under two phosphorus conditions: HP and LP.

	EMBRAPA048				TMG7063		
	WW		WS‐R5	WS‐V5 + R5	WW	WS‐R5	WS‐V5 + R5
*A*_max_ (μmol (CO_2_) m^−2^ s^−1^)	HP	20.36 ± 0.21 **Aa**	19.58 ± 0.34 **Aa**	21.14 ± 0.85 **Aa**	25.70 ± 1.26 **Aa**	21.43 ± 0.99 **Aa**	20.17 ± 1.46 **Aa**
	LP	9.42 ± 0.82 **Ba**	10.63 ± 0.49 **Ba**	12.80 ± 0.46 **Ba**	15.57 ± 3.26 **Ba**	9.04 ± 0.86 **Ba**	17.73 ± 1.87 **Ba**
*I*_max_ (μmol (photons) m^−2^ s^−1^)	HP	552.75 ± 8.46 **Aa**	505 ± 8.80 **Aa**	426.75 ± 10.28 **Aa**	530 ± 19.98 **Aa**	538 ± 2.46 **Aa**	529.66 ± 10.81 **Aa**
	LP	217.25 ± 19.57 **Ba**	227.25 ± 15.71 **Ba**	294.66 ± 2.78 **Ba**	411 ± 21.51 **Ba**	267.33 ± 9.07 **Ba**	438 ± 15.78 **Ba**
*I*_comp_ (μmol (CO_2_) μmol^−1^ (photons))	HP	50.60 ± 1.35 **n.s**.	20.17 ± 1.78 **n.s**.	28.01 ± 2.68 **n.s**.	24.74 ± 2.94 **n.s**.	23.11 ± 2.30 **n.s**.	28.97 ± 3.21 **n.s**.
	LP	37.26 ± 5.80 **n.s**.	22.50 ± 2.67 **n.s**.	32.97 ± 3.27 **n.s**.	38.08 ± 2.93 **n.s**.	34.68 ± 1.88 **n.s**.	34.7096 ± 1.65 **n.s**.
ϕ (*I*_comp_)	HP	0.053 ± 0.002 **A**	0.050 ± 0.002 **A**	0.058 ± 0.006 **A**	0.061 ± 0.004 **A**	0.054 ± 0.003 **A**	0.050 ± 0.002 **A**
	LP	0.084 ± 0.032 **B**	0.066 ± 0.008 **B**	0.061 ± 0.016 **B**	0.049 ± 0.006 **B**	0.038 ± 0.001 **B**	0.052 ± 0.003 **B**
*F*_v_/*F*_m_	HP	0.846 ± 0.002 **A**	0.841 ± 0.001 **A**	0.845 ± 0.0006 **A**	0.847 ± 0.0007 **Aa**	0.836 ± 0.003 **Ab**	0.837 ± 0.002 **Ab**
	LP	0.772 ± 0.004 **B**	0.777 ± 0.008 **B**	0.774 ± 0.016 **B**	0.805 ± 0.009 **Ba**	0.744 ± 0.021 **Bc**	0.782 ± 0.008 **Bb**
Total chlorophyll index	HP	47.98 ± 0.55 **Aa**	35.83 ± 2.11 **Ab**	40.71 ± 1.82 **Ab**	44.43 ± 0.67 **Aa**	34.80 ± 1.80 **Ab**	36.95 ± 1.41 **Ab**
	LP	39.98 ± 0.83 **Ba**	35.88 ± 1.35 **Bb**	36.87 ± 3.39 **Bb**	40.25 ± 1.19 **Ba**	29.38 ± 1.87 **Bb**	33.05 ± 3.04 **Bb**

*Note:* Different lowercase letters indicate significant differences by Tukey's test (*p* < 0.05) between water stress, and different uppercase letters indicate significant differences between phosphorus nutrition (in bold). Data are the mean and standard error of five repetitions. n.s. not significant.

**FIGURE 3:**
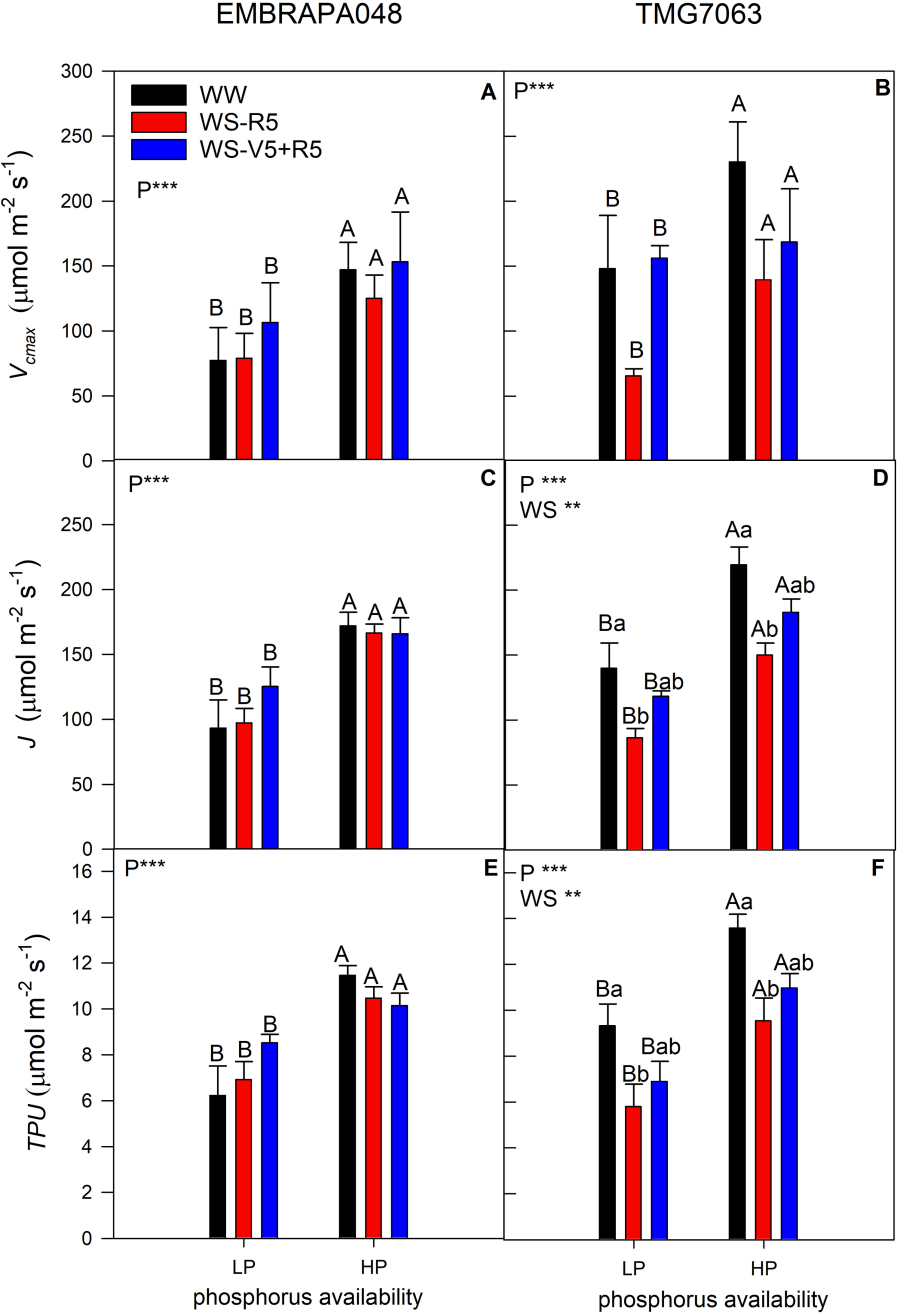
Maximum carboxylation rate (*V*_cmax_; A, B), electron transport capacity (*J*; C, D), triose phosphate utilization (*TPU*; E, F) in soybean cultivars, EMBRAPA048 (A, C, E) and TMG7063 (B, D, F) on the last day of severe water stress period under high P conditions (HP) or P‐deficient conditions (LP). The bars represent the mean of five repetitions, and the error bars represent the standard error of the mean. Only when significant by the ANOVA, the uppercase letters indicate statistical differences between nutritional conditions, and lowercase letters indicate statistical differences between water conditions, determined by Tukey's test at the 5% confidence level.

In both cultivars, P deficiency decreased the maximum Rubisco carboxylation rate (Figure 3A,B), electron transport rate (Figure 3C,D), and triose phosphate utilization (Figure 3E,F). While in the drought‐tolerant cultivar EMBRAPA048, the recovery from water deficit did not influence *V*_cmax_, *J*, and *TPU* (Figure 3), in the sensitive cultivar TMG7063, plants that were exposed to two cycles of water stress (WS‐V5 + R5) showed *J* (Figure 3D) and *TPU* (Figure 3F) higher than plants from WS + R5 and similar to WW plants.

The cultivar EMBRAPA048 showed lower mesophyll conductance under P deficiency conditions than under high P, resulting in greater mesophyll limitation (Table 2). Stomatal limitation occurred only under P deficiency conditions during the recovery period, and no significant differences were observed in biochemical limitation and respiratory rates for this same cultivar.

**TABLE 2:** Mesophyll conductance (*g*_m_), mesophyll limitation (*l*_m_), stomatal limitation (*l*_s_), biochemical limitation (*l*_b_), dark respiration (*R*_D_), photorespiration (*P*_r_) of soybean cultivars EMBRAPA048 and TMG7063 during the recovery period from water stress under two phosphorus conditions, HP and LP.

		EMBRAPA048			TMG7063		
		WW	WS‐R5	WS‐V5 + R5	WW	WS‐R5	WS‐V5 + R5
*g*_m_ (mmol m^−2^ s^−1^)	HP	0.19 ± 0.005 **A**	0.29 ± 0.057 **A**	0.23 ± 0.033 **A**	0.18 ± 0.025 **b**	0.12 ± 0.027 **b**	0.25 ± 0.058 **a**
	LP	0.04 ± 0.008 **B**	0.04 ± 0.006 **B**	0.06 ± 0.005 **B**	0.06 ± 0.015 **b**	0.04 ± 0.005 **b**	0.29 ± 0.003 **a**
*l*_m_	HP	0.30 ± 0.07 **B**	0.17 ± 0.05 **B**	0.29 ± 0.06 **B**	0.47 ± 0.04 **a**	0.45 ± 0.07 **a**	0.35 ± 0.08 **b**
	LP	0.61 ± 0.02 **A**	0.59 ± 0.03 **A**	0.60 ± 0.06 **A**	0.66 ± 0.03 **a**	0.60 ± 0.03 **a**	0.17 ± 0.007 **b**
*l*_s_	HP	0.29 ± 0.06 **A**	0.51 ± 0.10 **A**	0.37 ± 0.04 **A**	0.22 ± 0.01 **c**	0.23 ± 0.02 **c**	0.26 ± 0.02 **b**
	LP	0.10 ± 0.01 **B**	0.12 ± 0.02 **B**	0.10 ± 0.01 **B**	0.11 ± 0.002 **d**	0.12 ± 0.01 **d**	0.53 ± 0.005 **a**
*l*_b_	HP	0.40 ± 0.03 **n.s**.	0.30 ± 0.05 **n.s**.	0.32 ± 0.03 **n.s**.	0.29 ± 0.05 **n.s**.	0.30 ± 0.09 **n.s**.	0.38 ± 0.06 **n.s**.
	LP	0.28 ± 0.02 **n.s**.	0.2 ± 0.03 **n.s**.	0.28 ± 0.05 **n.s**.	0.21 ± 0.04 **n.s**.	0.26 ± 0.02 **n.s**.	0.22 ± 0.03 **n.s**.
*R*_D_ (μmol (CO_2_) m^−2^ s^−1^)	HP	1.91 ± 0.17 **n.s**	0.77 ± 0.13 **n.s**	1.68 ± 0.13 **n.s**	1.63 ± 0.05 **Aa**	0.78 ± 0.02 **Ab**	1.09 ± 0.09 **Ab**
	LP	1.11 ± 0.05 **n.s**	0.73 ± 0.02 **n.s**	1.33 ± 0.04 **n.s**	1.68 ± 0.07 **Aa**	0.94 ± 0.03**Ab**	0.9 ± 0.10 **Ab**
*P*_r_ (μmol (CO_2_) m^−2^ s^−1^)	HP	5.52 ± 0.67 **A**	5.74 ± 0.49 **A**	4.87 ± 0.23 **A**	6.68 ± 0.51 **A**	6.54 ± 0.62 **A**	5.43 ± 0.51 **A**
	LP	3.50 ± 0.40 **B**	4.08 ± 0.60 **B**	4.27 ± 0.12 **B**	4.76 ± 0.42 **B**	3.48 ± 0.45 **B**	4.22 ± 0.35 **B**

*Note:* Different lowercase letters indicate significant differences by Tukey's test (*p* < 0.05) between water deficit conditions, and different uppercase letters indicate significant differences between phosphorus nutrition conditions (in bold). Data are the mean and standard error of five repetitions. n.s. not significant.

On the other hand, cultivar TMG7063 decreased mesophyll conductance during recovery from water stress, regardless of the P nutritional condition. Plants of the cultivar TMG7063 subjected to both stress cycles (WS‐V5 + R5) exhibited higher mesophyll conductance and lower mesophyll limitation during the recovering phase when compared to plants from the other treatments. In contrast, plants subjected only to stress at R5 (WS‐R5) showed lower mesophyll conductance and consequently higher mesophyll limitation. During recovery from water deficit, the cultivar TMG7063 in the WS‐V5 + R5 treatment also maintained greater stomatal limitation in plants under P deficiency. Plants from the WS‐R5 treatment presented full recovery, with the lowest stomatal limitation values. In summary, while the WS‐R5 treatment resulted in mesophyll limitation to the soybean TMG7063 cultivar, plants from the WS‐V5 + R5 treatment exhibited stomatal limitation. The respiratory rate of cultivar TMG7063 was lower upon recovery from water stress, regardless of soil P availability (Table 2). There was no difference in respiratory rates in EMBRAPA048. The photorespiration rate was higher in plants from both cultivars under high P conditions, regardless of water stress (Table 2).

The grain dry mass of EMBRAPA048 cultivar was negatively impacted only by P deficiency. The grain dry mass of TMG7063 cultivar grown under high P conditions and subjected to WS‐R5 and WS‐V5 + R5 treatments was lower than control plants. However, this production was still higher than that of plants with P deficiency subjected to water stress (Table 3). The dry mass of leaves, roots, and total dry mass, P concentration in leaves at R5 and R8, were affected only by P deficiency in both cultivars (Table 3).

**TABLE 3:** Dry mass data of grain, dry mass of leaves, dry mass of roots, total dry mass, leaf area (LA), and phosphorus concentration of EMBRAPA048 and TMG7063 soybean cultivars during the recovery period from water deficit under two phosphorus conditions, HP and LP.

	EMBRAPA048				TMG7063		
	WW		WS‐R5	WS‐V5 + R5	WW	WS‐R5	WS‐V5 + R5
Grain (g DW Plant^−1^)	HP	37.95 ± 1.02 **A**	30.74 ± 2.32 **A**	28.68 ± 1.69 **A**	30.72 ± 2.69 **Aa**	22.23 ± 3.16 **Ab**	23.01 ± 2.74 **Ab**
	LP	6.82 ± 0.55 **B**	8.48 ± 0.75 **B**	7.17 ± 1.15 **B**	6.48 ± 0.81 **Ba**	4.24 ± 0.65 **Bb**	4.73 ± 0.39 **Bb**
Leaf (g DW Plant^−1^)	HP	5.42 ± 0.91 **A**	4.33 ± 1.00 **A**	2.85 ± 0.75 **A**	5.23 ± 0.50 **A**	3.31 ± 0.59 **A**	3.22 ± 0.62 **A**
	LP	1.09 ± 0.12 **B**	1.50 ± 0.10 **B**	1.49 ± 0.22 **B**	0.81 ± 0.33 **B**	0.62 ± 0.14 **B**	0.84 ± 0.13 **B**
Root (g DW Plant^−1^)	HP	20.86 ± 2.52 **A**	21.01 ± 1.89 **A**	20.05 ± 1.57 **A**	15.07 ± 2.66 **A**	11.40 ± 1.14 **A**	11.94 ± 1.56 **A**
	LP	6.88 ± 0.38 **B**	7.55 ± 0.14 **B**	5.79 ± 0.70 **B**	4.96 ± 0.77 **B**	4.17 ± 0.79 **B**	2.81 ± 0.27 **B**
Total (g DW Plant^−1^)	HP	103.42 ± 4.65 **A**	85.02 ± 6.16 **A**	77.52 ± 1.63 **A**	77.16 ± 7.33 **A**	63.56 ± 6.51 **A**	63.05 ± 4.18 **A**
	LP	21.43 ± 1.11 **B**	25.82 ± 1.59 **B**	21.36 ± 3.04 **B**	17.08 ± 2.33 **B**	13.84 ± 2.23 **B**	12.89 ± 1.16 **B**
LA (cm^2^)	HP	3356.79 ± 206.53 **A**	2589.54 ± 177.99 **A**	2561.20 ± 184.27 **A**	2275.75 ± 323.11 **A**	1624.02 ± 270.98 **A**	1729.56 ± 238.75 **A**
	LP	824.57 ± 43.63 **B**	994.36 ± 33.35 **B**	855.98 ± 233.89 **B**	558.57 ± 63.94 **B**	384.73 ± 94.46 **B**	494.31 ± 107.88 **B**
[P] on leaves R5 (dag kg^−1^)	HP	0.21 ± 0.008 **A**	0.22 ± 0.019 **A**	0.19 ± 0.011 **A**	0.21 ± 0.014 **A**	0.24 ± 0.004 **A**	0.23 ± 0.013 **A**
	LP	0.05 ± 0.0009 **B**	0.06 ± 0.002 **B**	0.07 ± 0.003 **B**	0.12 ± 0.007 **B**	0.11 ± 0.005 **B**	0.09 ± 0.002 **B**
[P] on leaves R8 (dag kg^−1^)	HP	0.12 ± 0.009 **A**	0.18 ± 0.008 **A**	0.18 ± 0.023 **A**	0.21 ± 0.035 **A**	0.12 ± 0.008 **A**	0.15 ± 0.012 **A**
	LP	0.04 ± 0.001 **B**	0.03 ± 0.0008 **B**	0.05 ± 0.001 **B**	0.08 ± 0.001 **B**	0.06 ± 0.002 **B**	0.07 ± 0.006 **B**

*Note:* Different lowercase letters indicate significant differences by Tukey's test (*p* < 0.05) between water stress, and different uppercase letters indicate significant differences between phosphorus nutrition (in bold). Data are the mean and standard error of five repetitions.

Soybean grain oil content, protein content, and oil composition were not affected by water deficit conditions in both cultivars, except for C18:1. Grain oil content was higher in EMBRAPA048 cultivar under P deficiency compared to HP conditions, whereas protein content was higher both in EMBRAPA048 and TMG7063 exposed to HP than LP conditions (Table 4). The cultivar EMBRAPA048 showed no change in palmitic acid (C16:0) and linolenic acids (C18:3), regardless of both P and water conditions. However, there was an increase in the proportion of stearic acid (C18:0) and oleic acid (C18:1), and a decrease in linoleic acid (C18:2) in HP‐treated plants compared to LP plants (Table 4). For the cultivar TMG7063, the unsaturated fatty acids [as oleic acid (C18:1) and linolenic acid (C18:3)] increased under HP conditions, while linoleic acid (C18:2) increased under LP conditions. In this cultivar, the saturated fatty acids palmitic acid (C16:0) and stearic acid (C18:0) were not altered in response to both treatments with different water and P availability conditions (Table 4).

**TABLE 4:** Oil content (%), protein content (%), and the fatty acids grain content (%) as palmitic acid (C16:0), stearic acid (C18:0), oleic acid (C18:1), linoleic acid (C18:2), and linolenic acid (C18:3) of soybean cultivars EMBRAPA048 and TMG7063 during the recovery period of water deficit under two phosphorus conditions, HP and LP.

	EMBRAPA048				TMG7063		
	WW		WS‐R5	WS‐V5 + R5	WW	WS‐R5	WS‐V5 + R5
Oil content (%)	HP	17.21 ± 0.51 **B**	17.34 ± 1.25 **B**	18.09 ± 0.62 **A**	15.71 ± 1.95 **n.s**.	18.31 ± 1.15 **n.s**.	18.54 ± 0.68 **n.s**.
	LP	19.40 ± 0.52 **A**	19.55 ± 0.40 **A**	17.09 ± 0.48 **B**	17.18 ± 0.94 **n.s**.	17.66 ± 1.02 **n.s**.	19.98 ± 0.96 **n.s**.
Protein content (%)	HP	33.94 ± 0.12 **A**	33.67 ± 1.38 **A**	35.24 ± 0.51 **A**	33.88 ± 1.06 **A**	32.80 ± 0.75 **A**	32.77 ± 0.41 **A**
	LP	29.48 ± 0.46 **B**	29.15 ± 0.27 **B**	30.62 ± 0.85 **B**	31.57 ± 0.69 **B**	28.94 ± 1.66 **B**	28.24 ± 1.85 **B**
C16:0	HP	12.19 ± 0.03 **n.s**.	11.90 ± 0.04 **n.s**.	11.71 ± 0.19 **n.s**.	12.52 ± 0.27 **n.s**.	12.33 ± 0.23 **n.s**.	11.62 ± 0.06 **n.s**.
	LP	11.35 ± 0.16 **n.s**.	11.78 ± 0.21 **n.s**.	11.73 ± 0.19 **n.s**.	12.21 ± 0.17 **n.s**.	12.14 ± 0.21 **n.s**.	11.86 ± 0.35 **n.s**.
C18:0	HP	3.91 ± 0.05 **A**	3.69 ± 0.07 **A**	4.07 ± 0.18 **A**	3.94 ± 0.12 **n.s**.	4.00 ± 0.13 **n.s**.	3.97 ± 0.08 **n.s**.
	LP	3.22 ± 0.09 **B**	3.24 ± 0.08 **B**	3.48 ± 0.20 **B**	3.73 ± 0.11 **n.s**.	4.01 ± 0.12 **n.s**.	4.32 ± 0.21 **n.s**.
C18:1	HP	23.73 ± 0.10 **Bb**	27.00 ± 0.59 **Aa**	26.11 ± 0.47 **Aa**	24.07 ± 1.09 **A**	25.90 ± 0.85 **A**	27.40 ± 0.95 **A**
	LP	23.71 ± 0.63 **Bb**	22.35 ± 0.31 **Bb**	22.55 ± 0.50 **Bb**	22.79 ± 0.46 **B**	22.89 ± 0.33 **B**	23.29 ± 0.28 **B**
C18:2	HP	54.15 ± 0.16 **B**	51.70 ± 0.64 **B**	52.39 ± 0.40 **B**	52.97 ± 0.89 **B**	51.07 ± 0.85 **B**	50.28 ± 0.48 **B**
	LP	56.13 ± 0.58 **A**	56.65 ± 0.49 **A**	56.57 ± 0.73 **A**	55.08 ± 0.15 **A**	54.96 ± 0.28 **A**	54.65 ± 0.31 **A**
C18:3	HP	6.00 ± 0.10 **n.s**.	5.69 ± 0.11 **n.s**.	5.70 ± 0.22 **n.s**.	6.48 ± 0.14 **A**	6.66 ± 0.44 **A**	6.71 ± 0.41 **A**
	LP	5.56 ± 0.18 **n.s**.	5.94 ± 0.04 **n.s**.	5.64 ± 0.24 **n.s**.	6.18 ± 0.10 **B**	5.98 ± 0.10 **B**	5.86 ± 0.15 **B**

*Note:* Lowercase letters indicate significant differences by Tukey's test (*p* < 0.05) between water stress conditions, and uppercase letters indicate significant differences between phosphorus nutrition (in bold). Data are the mean and standard error of five repetitions. n.s. not significant.

## 4 Discussion

Phosphorus deficiency had a lesser effect on the response to water deficit in the drought‐tolerant cultivar (EMBRAPA048). However, in the sensitive cultivar (TMG7063), P was critical to trigger a compensatory effect. The ability to recover physiological processes over time from drought can generally be divided into three main categories: partial, complete, and compensatory recovery (Xu et al. 2010), which are influenced by P nutrition and genotype. Surprisingly, plants under P deficiency that were exposed to two cycles of water deficit showed a greater capacity for photosynthetic recovery. Both cultivars showed complete recovery of *A*_max_, *I*_max_, effective quantum yield, respiratory rates, and *V*_cmax_. While the tolerant cultivar fully recovered mesophyll conductance, the sensitive cultivar showed compensatory recovery, which may have positively influenced the total recovery of *J* and *TPU*.

### 4.1 Photosynthetic Responses to Drought in Soybean Cultivars Under High and Low P

The cultivars showed a low capacity to synthesize photosynthetic pigments after resuming irrigation, resulting in only partial recovery of chlorophyll levels. Under water deficit conditions, nitrogen fixation decreases, directly affecting the synthesis of new chlorophyll molecules (de Freitas et al. 2022). Furthermore, P deficiency impaired the resumption of chlorophyll synthesis under water deficit due to impairments in the plant's energy metabolism.

Despite the lower total chlorophyll levels after resuming irrigation in the cultivar EMBRAPA048, the complete recovery of *F*_v_/*F*_m_ indicates an improved reorganization of the photosynthetic apparatus even under P deficiency. The unchanged *F*_v_/*F*_m_ demonstrates a greater resistance of PSII to water deficit in the tolerant cultivar, indicating better adjustment in energy partitioning between photosystems and photoprotective energy dissipation (Kalaji et al. 2016). The sensitive cultivar under P deficit exhibited a more effective investment in restoring PSII integrity in the WS‐V5 + R5 treatment, which resulted in higher *F*_v_/*F*_m_ values compared to plants exposed to only one water deficit cycle (WS‐R5). The drought‐sensitive soybean cultivar exposed to a first moderate water deficit condition increased its ability to resist future stress events, accelerating the recovery process and consequently mitigating the damage (Bester et al. 2024).

The diffusive phase of photosynthesis is a fundamental process that regulates gas exchange between plants and the environment (Flexas and Medrano 2002; Pilon et al. 2018). In addition to the lower stomatal conductance, the decrease in mesophyll conductance contributed to the reduction in chloroplastidic CO_2_ concentration, causing a decrease in the photobiochemical stage parameters. The reduction in stomata density and size is also a common effect of P deficiency (Khan et al. 2023), and may have contributed to the decrease in CO_2_ uptake. However, in our study, the main cause of decreased *A*_n_ uptake was the restriction of internal CO_2_ concentration in leaf mesophyll, which caused biochemical limitations due to lower instantaneous carboxylation efficiency, followed by stomatal limitation. Biochemical processes were impaired by P deficiency, leading to a decrease in ATP and NADPH production and lower regeneration of ribulose‐1,5‐bisphosphate (Medina et al. 2022). Therefore, the values of *V*_cmax_, *J*, and *TPU* were even lower under P deficiency and water deficit.

The temporal dynamics of stomatal closure during water deficit depend on the plant's ability to establish a threshold between maintaining carbon fixation and regulating the water supply capacity of the hydraulic systems to avoid embolism (Volaire 2018). The drought‐tolerant cultivar showed a greater capacity to regulate stomatal opening, so the leaf water potential was higher even under P deficiency.

### 4.2 Proline and Reactive Oxygen Species Control in Soybean Cultivars Under Drought and P‐Deficiency Stress

Proline is an amino acid that protects plants from various stress conditions and also helps plants recover faster from stress (Al‐Karaki et al. 2012; Ben Ahmed et al. 2010; Medina et al. 2023). Plants constantly synthesize reactive oxygen species as by‐products of various metabolic pathways. However, under water deficit, ROS concentration may exceed the capacity of antioxidant metabolism to eliminate them.

Excessive ROS production leads to significant oxidative damage to proteins, DNA, and lipids, thereby inhibiting plant growth. Some reports indicate that proline eliminates ROS and other free radicals (Ben Ahmed et al. 2010; Sperdouli and Moustakas 2012; Szabados and Savouré 2010). By maintaining high proline levels in leaf tissue after water deficit, the cultivar EMBRAPA048 may have favored its antioxidant metabolism, which is confirmed by the lower H_2_O_2_ levels. Since the cultivar TMG7063 did not significantly increase proline concentration upon recovery from water deficit, H_2_O_2_ production was higher than that of cultivar EMBRAPA048, especially under P deficiency.

### 4.3 Plant Biomass, Production, and Grain Quality in Soybean Cultivars Under P Deficiency

The suppression of photosynthesis under drought, combined with P deficiency, decreases ATP production, resulting in less energy for plant growth and development. This was evident through the primary effect of P deficiency on root, leaf, and total biomass, as well as decreases in grain production and quality in both soybean cultivars. Our study shows that P deficiency leads to losses in leaf area and biomass. However, the drought‐tolerant cultivar was able to produce more biomass than the sensitive cultivar. This demonstrates that the mechanism of drought tolerance may favor grain production under P deficiency. Studying the morphological characteristics of the plants can help researchers determine the effects of water deficit and rehydration on soybeans, as well as the changes in their patterns (Dong et al. 2019). However, despite pot experiments, as used in our study, being essential for controlled evaluations of stress responses, they may not fully capture the interactions between P nutrition and root development, limiting the extrapolation of results to production systems.

Soybeans are an important source of protein and vegetable oil. Only P deficiency had a negative effect on grain composition, as P is directly related to the formation of energy molecules used in N metabolism (Jin et al. 2006). This also explains the decrease in protein content in both soybean cultivars under P deficiency and also contributed to limited RubP regeneration in photosynthesis (Figure 3), impairing growth and yield. In addition to the decrease in protein content in EMBRAPA048, the oil content also increased. Fatty acid metabolism was stimulated under P deficiency. Linoleic acid (C18:2) contributed to this increase and impaired grain quality in both cultivars, and also linolenic acid (C18:3) in the drought‐sensitive cultivar, TMG7063. This lower quality would increase due to oil rancidity driven by the oxidation of polyunsaturated fatty acids after harvest (Gharby et al. 2025). With the growing interest in removing trans fatty acids from the diet, soybean breeders must focus on developing varieties that produce seeds with high levels of desirable fatty acids, such as oleic acid (Yin et al. 2016). However, its content was lower under P deficiency in both cultivars.

Optimizing P fertilization can enhance soybean nutrient use and photosynthetic efficiency while decreasing environmental impacts. Our study indicates that controlled water deficits during vegetative growth may prepare plants for better recovery during future droughts, supporting stable yields and desirable grain composition. These findings collectively promote integrated P and water management as a strategy for sustainable soybean production in changing climates.

## 5 Conclusion

P deficiency prolonged the decline in photosynthesis in response to water deficit in both cultivars. However, photosynthetic rates recovered faster under P deficiency than under high P conditions.

P deficiency had no effect on the response pattern to water deficit stress in the tolerant cultivar, but was crucial for triggering a compensatory effect on the sensitive cultivar.

The plants of the sensitive cultivar that were exposed to two cycles of deficit water showed compensatory effects in the recovery of mesophyll conductance and *F*_v_/*F*_m_, which were reflected in higher rates of *V*_cmax_, *J*, and *TPU*. However, the compensatory photosynthetic adjustments did not result in better grain production.

Grain quantity and quality were affected by P deficiency in both cultivars. P deficiency reduced the protein content and increased the oil content in the grains, especially the unsaturated fatty acids.

The physiological plasticity of genotypes susceptible to water deficit is impaired by P deficiency.

## Author Contributions

I.R.M.S. participated in the conduction, biochemical analysis, data analysis, and writing. G.H.R. participated in the conduction of the analyses and biochemical analysis. G.P.M.‐A. participated in the analysis of fatty acids. E.G.P. participated in the elaboration, data analysis, and writing.

## Funding

This work was supported by Conselho Nacional de Desenvolvimento Científico e Tecnológico, 305376/2023‐3, 406042/2022‐5.

Coordinação de Aperfeiçoamento de Pessoal de Nível Superior, 1.

## Data Availability

The datasets generated during the current study are available from the corresponding author on reasonable request.
